# Evocación histórica: Aniversario 80 años de la Revista de la Facultad de Ciencias Médicas de Córdoba

**DOI:** 10.31053/1853.0605.v80.n4.43234

**Published:** 2023-12-26

**Authors:** Norma Acerbi Cremades

**Affiliations:** 1 Prof. Consulto. Facultad de Ciencias Médicas. Ex Directora del Museo Histórico Hospital Nacional de Clínicas. UNC Universidad Nacional de Córdoba.

## Evocación histórica

Digno broche de oro para cerrar el año 2023, es el justo homenaje a la publicación de la **Revista de la Facultad de Ciencias Médicas de Córdoba**, en sus 80 años de magnífica trayectoria.

Es un honor para mí, poderme unir a la voz, cálida y meditada de los participantes de la presente publicación.

La idea de creación de la Revista FCM, surgió de dos mentes humanistas, ilustradas y progresistas: los Profesores Doctores León Sebastián Morra y Vicente J. Bertola, Decano y Vice Decano, respectivamente de la Facultad de Ciencias Médicas.UNC.

Ambos estaban seguros que una Revista científico-intelectual, constituiría una fuente original, de conocimientos y enseñanzas para el país y el extranjero. Sería el trabajo viviente y expresivo de la capacidad de nuestros profesionales, además de una magnífica obra de extensión universitaria.

El HCD, en sesión del 30 de Marzo de 1943, aprobó el Proyecto del Sr. Decano, Prof. Dr. León S. Morra, disponiendo:

Art. 1-Creáse la Revista de la Facultad de Ciencias Médicas, encargada de la publicación y difusión de trabajos de los Profesores y personal técnico de las distintas Escuelas y de las Investigaciones realizadas por sus Institutos y Laboratorios.

Art. 2-La publicación contendrá también una breve reseña del movimiento administrativo de la Facultad, como igualmente un extracto de las Actas de las sesiones que celebre el HCD.

Art. 3-Será dirigida por una Comisión de cinco Profesores; tres de Medicina; uno de Odontología y uno de Farmacia y Bioquímica, designados por el HCD.

Art. 4-Tendrá a su frente un Director; un Secretario de Redacción y algún otro personal que anualmente se juzgue necesario. Contarán con la cooperación de la Secretaría y demás dependencias de la FCM.

Art. 5-Será una publicación semestral hasta que se le pueda dar un carácter de mayor frecuencia. El Director de la Revista será designado por el Sr. Decano, previo acuerdo del HCD, en cambio el Secretario de Redacción y demás personal, serán designados directamente por el Sr. Decano.

Art. 6-Se solicitará al HCS, la encuadernación e impresión de la Revista, en los Talleres Gráficos de la Universidad.

El día 1 de Julio de 1943, se firmó el Decreto N° 3533, el que **Reglamentó el Funcionamiento** de la publicación.

Este Decreto contiene 15 Artículos, de los que me permito recordar alguna de sus disposiciones, tales como:

a) Será una publicación trimestral, con no más de 130 páginas.

b) El Director será por lo menos un Profesor Adjunto, con ocho años de antigüedad en el cargo.

c) Se podrán aceptar fondos provenientes de suscripciones, avisos publicitarios, donaciones, otros, los que percibidos por el Director, serán depositados en la Tesorería de la UNC, en Cuenta Conjunta y destinados a reforzar la partida aplicada a la publicación de la Revista.

d) La Revista dependerá del Decanato de la FCM.

Solo en el primer Volumen de la obra, aparece el movimiento administrativo de la FCM; la nómina de Profesores de la Casa y el número de clases, dictadas por los mismos (Art. 2 del Proyecto),

En los Volúmenes de las Décadas del 40 y 50, aparece publicidad de importantes firmas comerciales, (Art. 13 del Reglamento de funcionamiento), las que ya desaparecen en la Década del 60.

Desde el año 2009, por Resolución Decanal, la Revista FCM, fue incorporada a la esfera de la Secretaria de Ciencia y Tecnología de la FCM-UNC.

Es interesante recordar la nómina de Directores, aunque sin citar nombres, todos Profesores de verdadera intelectualidad, con un sentido cabal de sus deberes y una clara visión del porvenir.

En muchos casos, dejaron la impronta de sus personalidades puestas de manifiesto en la renovación de la fisonomía de la Revista por ejemplo:

En la conformación del Directorio y /o de los Comités Editorial y de Redacción; en el ordenamiento de los trabajos o en el diseño de sus tapas e interior, por supuesto siempre, con la venia del Sr. Decano y su HCD y acorde con las necesidades y circunstancia de cada periodo.

En un comienzo, según lo estipulaba el Art. 5 del Proyecto, la Revista estaba dirigida por un Director; un Secretario de Redacción y una Comisión de cinco Profesores de las Escuelas. Medicina; Odontología; Farmacia y Bioquímica.

En la Década del 70 y 80, tiene un Director Editorial Jefe; un Comité de Redacción con cuatro Miembros y un Comité Editorial, con once Miembros.

En la Década del 90, a la figura del Director, se sumaron tres Directores Asociados; un Secretario Ejecutivo; el Comité de Redacción con diez Miembros y un Comité Editorial, con veintidós Miembros.

En el Siglo XXI, la Revista FCM, tiene un Director; tres Directores Honorarios; un Secretario Ejecutivo; un Comité de Redacción con doce Miembros y un Comité Editorial con setenta y cinco Miembros, todos Profesores Titulares de las diferentes Cátedras de la Facultad de Ciencias Médicas.

En una Década posterior, tanto el Comité de Redacción como el Comité Editorial, quedaron conformados con solo doce Miembros, nacionales y prestigiosos Especialistas del extranjero

La **Revista de la Facultad de Ciencias Médicas de Córdoba**, no podía permanecer ajena a la vorágine de cambios en las estructuras científico-técnico –tecnológicas, en las que vivimos. Hizo su presentación digital desde el año 2012, permitiendo, con un sentido más abarcativo, la información a profesionales, docentes, investigadores y alumnos, nacionales y extranjeros, interconectados por Internet, en forma tal que sus creadores jamás hubieran imaginado.

La tecnología digital, sin duda, es un gran recurso que mantiene el diálogo entre los autores de trabajos y la sociedad universitaria que los consulta.

Desde el año 2000, la Revista FCM, agregó un Suplemento con los Resúmenes de los trabajos presentados en las "Jornadas de Investigación Científica de la Facultad de Ciencias Médicas-UNC".

Dichas Jornadas se repiten anualmente con gran éxito.

Las Jornadas de Investigación Científica, son la expresión, como indica su nombre, de las investigaciones, aportes e inquietudes, de los diferentes Institutos, Laboratorios y Cátedras de la Facultad de Ciencias Médicas.

En las páginas de la Revista, por medio del Suplemento, los lectores pueden valorar la inteligencia, la responsabilidad, entusiasmo y esfuerzo de nuestros investigadores.

Hace ya tiempo, es decir 80 Años, que la vida de la **Revista de la Facultad de Ciencias Médicas de Córdoba**, nacida de una idea generosa, ha ido creciendo, alimentada, animada, apoyada y adaptada por los Directivos, en las diferentes épocas, representando los principios y vivencias de nuestra Universidad.

En cada año que pasa, se produce el milagro de la conjunción de dos generaciones, la de los verdaderos Maestros, con la de los verdaderos Discípulos.

Sin duda, que esa provechosa conjunción de valores, mantendrá viva la excelencia, la continuidad y permanencia de la existencia de la **Revista de la Facultad de Ciencias Médicas de Córdoba**, de la Universidad Nacional de Córdoba.

Prof. Consulto Dra. Norma Acerbi Cremades

